# Unravelling reference bias in ancient DNA datasets

**DOI:** 10.1093/bioinformatics/btae436

**Published:** 2024-07-03

**Authors:** Stephanie Dolenz, Tom van der Valk, Chenyu Jin, Jonas Oppenheimer, Muhammad Bilal Sharif, Ludovic Orlando, Beth Shapiro, Love Dalén, Peter D Heintzman

**Affiliations:** Centre for Palaeogenetics, Svante Arrhenius väg 20C, Stockholm, SE-106 91, Sweden; Department of Geological Sciences, Stockholm University, Stockholm, SE-106 91, Sweden; Centre for Palaeogenetics, Svante Arrhenius väg 20C, Stockholm, SE-106 91, Sweden; Department of Bioinformatics and Genetics, Swedish Museum of Natural History, Stockholm, SE-114 18, Sweden; Science for Life Laboratory, Stockholm, SE-171 65, Sweden; Centre for Palaeogenetics, Svante Arrhenius väg 20C, Stockholm, SE-106 91, Sweden; Department of Bioinformatics and Genetics, Swedish Museum of Natural History, Stockholm, SE-114 18, Sweden; Department of Zoology, Stockholm University, Stockholm, SE-106 91, Sweden; Department of Biomolecular Engineering, University of California Santa Cruz, Santa Cruz, CA, 95064, United States; Centre for Palaeogenetics, Svante Arrhenius väg 20C, Stockholm, SE-106 91, Sweden; Department of Zoology, Stockholm University, Stockholm, SE-106 91, Sweden; Centre for Anthropobiology and Genomics of Toulouse (CAGT, CNRS UMR5288), University Paul Sabatier, Faculté de Santé, Toulouse, 31000, France; Department of Ecology and Evolutionary Biology, University of California Santa Cruz, Santa Cruz, CA, 95064, United States; Howard Hughes Medical Institute, University of California Santa Cruz, Santa Cruz, CA, 95064, United States; Centre for Palaeogenetics, Svante Arrhenius väg 20C, Stockholm, SE-106 91, Sweden; Department of Bioinformatics and Genetics, Swedish Museum of Natural History, Stockholm, SE-114 18, Sweden; Department of Zoology, Stockholm University, Stockholm, SE-106 91, Sweden; Centre for Palaeogenetics, Svante Arrhenius väg 20C, Stockholm, SE-106 91, Sweden; Department of Geological Sciences, Stockholm University, Stockholm, SE-106 91, Sweden

## Abstract

**Motivation:**

The alignment of sequencing reads is a critical step in the characterization of ancient genomes. However, reference bias and spurious mappings pose a significant challenge, particularly as cutting-edge wet lab methods generate datasets that push the boundaries of alignment tools. Reference bias occurs when reference alleles are favoured over alternative alleles during mapping, whereas spurious mappings stem from either contamination or when endogenous reads fail to align to their correct position. Previous work has shown that these phenomena are correlated with read length but a more thorough investigation of reference bias and spurious mappings for ancient DNA has been lacking. Here, we use a range of empirical and simulated palaeogenomic datasets to investigate the impacts of mapping tools, quality thresholds, and reference genome on mismatch rates across read lengths.

**Results:**

For these analyses, we introduce AMBER, a new bioinformatics tool for assessing the quality of ancient DNA mapping directly from BAM-files and informing on reference bias, read length cut-offs and reference selection. AMBER rapidly and simultaneously computes the sequence read mapping bias in the form of the mismatch rates per read length, cytosine deamination profiles at both CpG and non-CpG sites, fragment length distributions, and genomic breadth and depth of coverage. Using AMBER, we find that mapping algorithms and quality threshold choices dictate reference bias and rates of spurious alignment at different read lengths in a predictable manner, suggesting that optimized mapping parameters for each read length will be a key step in alleviating reference bias and spurious mappings.

**Availability and implementation:**

AMBER is available for noncommercial use on GitHub (https://github.com/tvandervalk/AMBER.git). Scripts used to generate and analyse simulated datasets are available on Github (https://github.com/sdolenz/refbias_scripts).

## 1 Introduction

The availability of ancient DNA (aDNA) sequence data has revolutionized our understanding of evolutionary processes and natural history (Green *et al.* 2010, van der Valk *et al.* 2021) but also poses analytical challenges due to the degraded nature of aDNA and the presence of environmental contaminants (Orlando *et al.* 2021). Further, as new boundaries for aDNA recovery beyond the million year time range are tested (van der Valk *et al.* 2021, Kjær *et al.* 2022, Fernandez-Guerra *et al.* 2023), the ability to robustly study increasingly damaged samples from diverged populations requires an assessment of current alignment tools, as target data become more diverged from reference genomes. Presently, the most common mapping tools for aDNA reads are BWA-aln (also known as BWA-backtrack), Bowtie2, and BWA-mem, each with applied aDNA parameters (Poullet and Orlando 2020, Oliva *et al.* 2021) and post-filtering map quality (MQ) scores of generally ≥25. Without employing proper data quality control checks, downstream analyses and the reliability of the results obtained from ancient genomes can become significantly biassed (Günther and Nettelblad 2019, Orlando *et al.* 2021).

Important summary statistics have been developed to assess aDNA data authenticity and quality, including post-mortem DNA damage patterns (Green *et al.* 2010, Jónsson *et al.* 2013, Skoglund *et al.* 2014), fragment length distributions (Green *et al.* 2010), the breadth of genome coverage (Pochon *et al.* 2023), and sequence read mapping biasses (van der Valk *et al.* 2021). Although a suite of bioinformatic tools to assess aDNA data have been developed, limitations remain, as none of the existing tools provide a comprehensive overview of all the statistics described above, thus requiring the use of multiple different tools, scripts, and separate computational runs to obtain the necessary information.

To address these limitations, we introduce a novel command-line based bioinformatics tool, AMBER (Assessing Mapping Biases and Evaluating Read Reliability). Further, we present the capability of AMBER to observe the impact of reference bias (preferential mapping of reads containing reference over alternative alleles), spurious mappings (mapping of reads that did not originate from that position in the genome), contamination, and the degree of divergence of mapped reads to the reference genome, in both simulated and empirical datasets. We further showcase the usage of AMBER with metagenomic datasets and within-individual comparisons of different genomic loci (Supplementary Texts S1 and S2; Supplementary Figs S1 and S2).

### 1.1 Tool description

AMBER is specifically designed for the quality assessment of aDNA sequence data directly from BAM-files, allows up to six samples to be analysed together, and eliminates the need for additional file formats, datasets, or preprocessing steps. This user-friendly software provides comprehensive insights into the quality of aDNA data by offering several key functionalities. Firstly, AMBER computes base mismatches between reads and the reference, normalized by the read length. This allows the user to identify biases introduced during read trimming, merging, mapping and quality filtering, and to determine appropriate sample-specific read length cutoffs in order to mitigate such biases (van der Valk *et al.* 2021). Secondly, AMBER assesses aDNA damage patterns by calculating the rate of C-to-T substitutions across sequence reads. An excess of these substitutions arise near read termini due to the accumulation of post-mortem cytosine deamination (Briggs *et al.* 2007). By characterizing damage patterns, users can distinguish authentic aDNA from modern contaminants, thereby ensuring the integrity of the dataset (Jónsson *et al.* 2013, Skoglund *et al.* 2014). AMBER also includes measures of DNA damage specifically at CpG sites, thereby allowing for the authentication of aDNA libraries that are chemically treated to eliminate cytosine deamination damage patterns outside of CpG sites (USER-treatment) (Briggs *et al.* 2010). Thirdly, AMBER outputs the aDNA fragment length distribution, a critical aspect for assessing the DNA degradation profile. The fragment length distribution helps users to evaluate the extent of DNA fragmentation, estimate the average size of endogenous DNA molecules, and assess the suitability of the data for further downstream analyses. Finally, AMBER provides an estimation of the genome coverage, by measuring both the fraction of the target genome covered by sequence reads (breadth of coverage) and the average sequence depth across the genome. These metrics aid the user in determining the representativeness and completeness of their genomic data, guiding downstream analyses such as variant calling, genomic sex determination, or the authentication of the presence of species of interest in metagenomic data (Supplementary Texts S1 and S2; Supplementary Figs S1 and S2). The running time of AMBER is ∼5–100× faster than other currently available software (Supplementary Text S3; Supplementary Table S1).

To demonstrate the utility of AMBER, we applied this new tool to multiple ancient genomes from diverse samples of different quality and age. Through these case studies, we showcase the capabilities of AMBER in assessing the quality of aDNA data and provide insights into different biases that can be introduced during the bioinformatic processing of ancient genomes.

## 2 Materials and methods

### 2.1 AMBER tool implementation

AMBER runs entirely in python3, with the only dependencies being *pysam* (Heger *et al.* 2014) and *matplotlib* (Hunter 2007). To run AMBER, a file containing the paths to a maximum of six BAM-files is provided by the user. AMBER calculates four different ancient DNA relevant statistics (described hereafter) and outputs these in a four-panelled plot. Optional parameters allow for the exclusion of specified contigs/scaffolds/chromosomes, the inclusion of error bars, and the plotting of fragment length distributions and genomic coverage by read count (recommended for within-sample technical comparisons).

#### 2.1.1 Edit distance by read length

AMBER records the length and edit distance (derived from the NM tag in the BAM-file) for each mapped read, following the strategy of (van der Valk *et al.* 2021). The average edit distance by read length is then plotted for all read lengths. Reads >300 bp in length are merged into the 300 bp bin and reads containing deletions or indels, reads that are clipped, or reads containing unknown bases (‘N’ characters) are excluded from the calculation.

#### 2.1.2 Post-mortem DNA damage

The reference sequence in the region of the mapped read is first reconstructed by AMBER using the MD tag in the BAM-file. Next, CG to TG (CpG sites) and all other C-to-T (non-CpG sites) mutations with respect to the reference sequence are recorded in a hash table together with their position in the read. All other mutations are recorded as ‘other’. The fraction of substitutions out of the total possible substitutions are then plotted with respect to their position in the read and averaged over all reads. This procedure follows the same strategy as in (Skoglund *et al.* 2014).

#### 2.1.3 Fragment length distribution

The lengths of all mapped read sequences are recorded in a hash table and subsequently the fraction of reads by read length are plotted as a line plot with shades.

#### 2.1.4 Genome coverage in 1000 bp windows

AMBER divides the reference genome into non-overlapping windows of 1000 bp in length, and for each window in the reference, the total read depth of the sample is recorded and subsequently saved into a hash table. An average read depth histogram is then plotted, with all windows above five times the average genome-wide coverage merged into the five times average coverage bin. A dashed vertical bar is plotted at the average read depth, calculated for all windows below five times the genome wide average. The breadth of coverage is shown by the fraction of the genome outside of the lowest depth bin (i.e. ∼0× coverage).

### 2.2 Evaluating AMBER and the impacts of reference bias

#### 2.2.1 Empirical datasets and initial data processing

We downloaded the raw sequencing data from seven previously published aDNA datasets, which included six palaeogenomes (one human, four faunal, one floral) and one ancient metagenome (Supplementary Table S2). We performed adapter trimming and read merging using fastp-v0.23.2, and removed reads below a length of 20 bp (or 30 bp in the cases of American mastodon and Siberian unicorn) and those that could not be merged (Chen *et al.* 2018).

#### 2.2.2 Empirical palaeogenomic sequence data processing

We mapped the empirical palaeogenomic datasets against either the Asian elephant (NCBI RefSeq GCF_024166365.1) with a human genome as a decoy (Feuerborn *et al.* 2020), Black rhinoceros (GCA_020826845.1), human (GRCh38.p14), horse (EquCab3; GCA_002863925.1), or maize (GCA_024505845.1) reference genomes (Supplementary Table S2). Mapping was done using three alignment algorithms: *BWA-aln* v0.7.17 (Li and Durbin 2009) with ancient DNA-specific parameters (-l 16500 -n 0.01 -o 2) (Green *et al.* 2010), *Bowtie2* v2.3.5.1 (Langmead and Salzberg 2012) on the --sensitive setting in the default end-to-end mode as recommended for ancient samples (Poullet and Orlando2020), or *BWA-mem* v0.7.17 (Li 2013) with ancient parameters (-t 8 -k 19 -r 2.5) (Xu et al., 2021). We note that *BWA-mem* is unable to align reads <30 bp. We further performed a limited comparison between three *Bowtie2* settings: (i) *end-to-end --sensitive*, (ii) *end-to-end --very-sensitive*, and (iii) *local --sensitive* (see Supplementary Text S7; Supplementary Fig. S6). We removed duplicate sequences using samtools rmdup v1.17 (Li and Durbin 2009) and applied an MQ filter of either 1, 20, 25, or 30.

#### 2.2.3 Simulated datasets and data processing

We simulated sequencing reads from the Asian elephant, Black rhinoceros, human, and a concatenation of all bacterial reference genomes (Genome Taxonomy Database [GTDB; June-2023 release]) using Gargammel (Renaud *et al.* 2017). A total of 18.1 million reads were simulated per reference genome with 100 000 reads sampled per read length bin from 20 to 200 bp. For the elephant and rhinoceros, each simulation was generated with either no divergence or a mean sequence divergence of 1% to 15% at 1% intervals for 1%–6% and 3% intervals from 6% to 15%, for a total of 10 simulated datasets per reference. Introduced divergence consisted of random mismatches across the length of reads. We therefore did not model an aDNA damage profile or consider heterogeneity in evolutionary rate across the genome. The human and bacterial reference genomes were simulated without divergence. Four libraries per divergence level were then constructed; (100% endogenous), (50% endogenous, 49% bacteria, 1% human), (10% endogenous, 89% bacteria, 1% human), and (1% endogenous, 98% bacteria, 1% human). Mapping, filtering, and analysis of the simulated reads followed the methodology given in Section 2.2.2, with the simulated elephant and rhinoceros datasets respectively mapped to their genome of origin. The proportion of mismapped reads was calculated by searching for reads whose mapping coordinates did not match their known coordinates of origin that were retained in the read header by Gargammel.

## 3 Results

### 3.1 Overview of datasets used to evaluate AMBER and investigate reference bias

We tested AMBER on a set of seven empirical aDNA datasets, together with datasets simulated from two faunal reference genomes. To demonstrate the versatility of AMBER, the empirical datasets were chosen to represent variations in species (human, faunal, floral, bacterial), sample age (2 ka - 2 Ma), endogenous content (3%–70%), genome coverage (0.3–42.4×), and sample type (tissue and sediment) (Supplementary Table S2). We additionally chose representatives that had undergone USER-treatment to remove aDNA damage, and taxa that could be mapped to a conspecific or required an interspecific reference genome. To verify and explore the drivers of observed reference biases, we generated a total of 80 simulated Asian elephant and Black rhinoceros genomic datasets. We constructed simulated libraries with either 100%, 50% 10%, or 1% endogenous DNA (elephant or rhino), and introduced random mismatches into the endogenous DNA , resulting in 10 datasets of 0%–15% divergence from the reference for each simulated library. We report simulated data from the elephant here and provide plots from both simulated genomes in Supplementary Data S1 and S2.

### 3.2 Estimating optimal minimum read length cutoffs

Ancient DNA typically consists of short fragments in the range of 10–150 bp (Sawyer *et al.* 2012). For highly degraded samples, the incorporation of ultrashort reads (≤35 bp) in downstream analysis can greatly increase the available genomic information. However, the inclusion of ultrashort reads increases the risk of spurious mismappings of endogenous reads and non-endogenous (environmental) contamination. Determining the optimal read length cutoff at which the amount of obtained genetic information is maximized while spurious mappings are minimized is sample-specific and relies on factors such as the DNA fragment length distribution, the proportion of endogenous DNA, and the sequence divergence between the sample and the reference genome, with the latter factor enhanced by aDNA damage. To avoid false inferences, aDNA studies often adopt a conservative minimal read length filter of 35 bp, potentially excluding a substantial portion of usable data (de Filippo *et al.* 2018, van der Valk *et al.* 2021).

**Figure 1.**
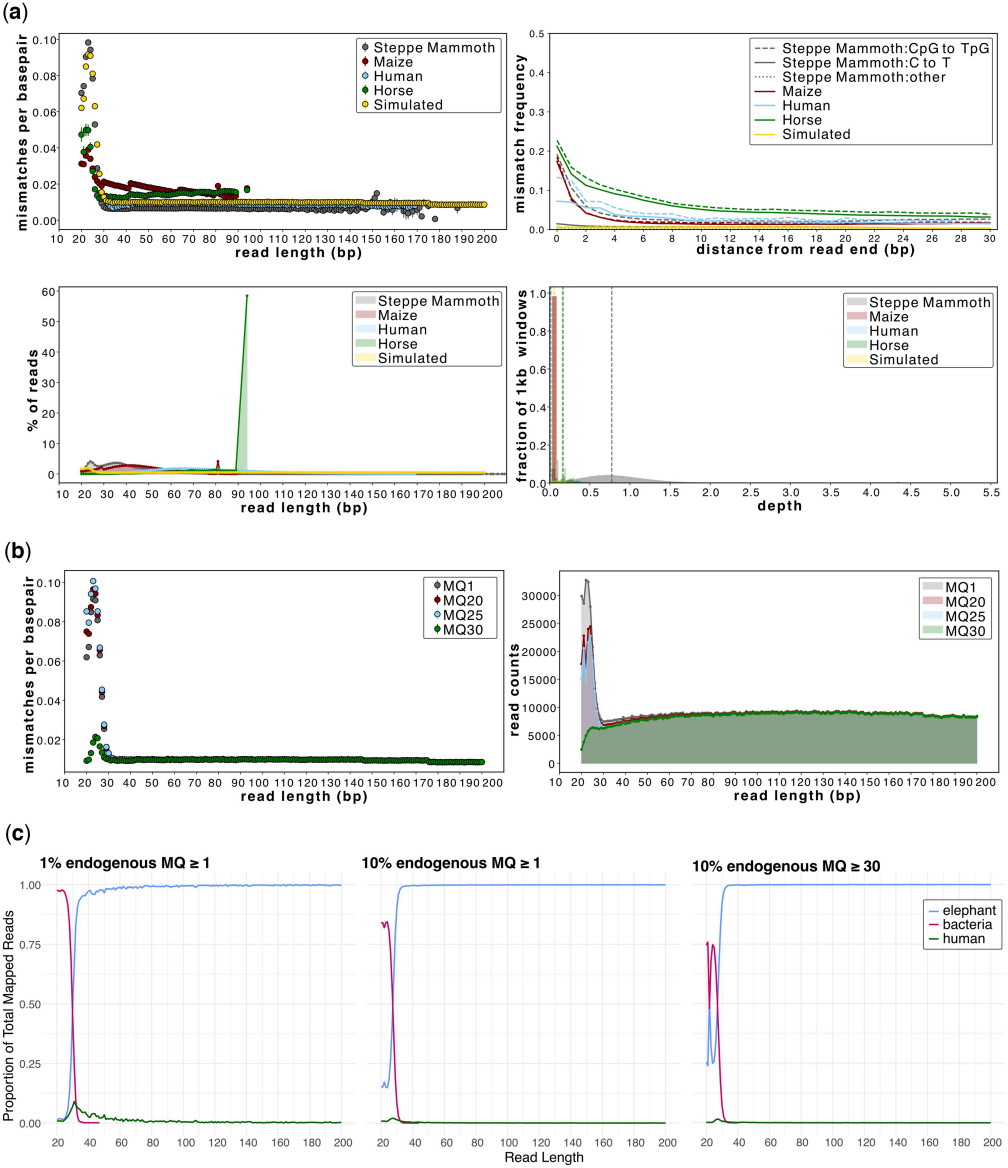
Assessing minimum read length thresholds in ancient genomic datasets aligned using *BWA-aln*. (a) AMBER plots at MQ ≥1 for four empirical datasets (steppe mammoth, maize, human, and horse) and a simulated dataset (10% endogenous elephant with 1% sequence divergence) showing a secondary peak at 20–30 bp; (b) mismatch and fragment length distribution plots for a simulated dataset of 10% endogenous elephant with 1% sequence divergence at four MQ thresholds; (c) the proportions of simulated endogenous elephant with 1% sequence divergence, human, and bacterial data aligned using *BWA-aln* to the Asian elephant genome for two endogenous DNA contents: 10% (MQ ≥1, ≥30) and 1% (MQ ≥1). Aligned ultrashort reads (≤35 bp) are dominated by bacteria, with this trend enhanced at a lower endogenous DNA content and only marginally reduced at MQ ≥30. For all comparisons, see Supplementary Data S3. In (c), zero values are not plotted

AMBER facilitates the evaluation of sample-specific read length cutoffs by plotting the mismatch rate between the mapped reads and the reference genome as a function of read length while simultaneously providing an overview of the overall read length distribution (Fig. 1). Without underlying bioinformatic biases, the average mismatches per base pair between a mapped read and the reference are expected to be independent of read length, with deviations from this expectation signalling erroneous mappings and reference bias. AMBER visualizes these deviations and allows the user to make an informed decision on selecting sample-specific read length cutoffs, thus enhancing the accuracy and usable yield of aDNA data (Fig. 1). To showcase this feature, we ran AMBER on human, steppe mammoth, horse, and maize genomes mapped with the *BWA-aln* algorithm and filtered for MQ ≥1, but note that this feature is also observed with other aligners and MQ thresholds (e.g. Section 3.4). We find a secondary peak in the fragment length distribution and that mismatch rates spike for reads below a length of 30 bp, suggesting that these ultrashort reads are enriched for spurious mappings and should be discarded from downstream analysis (Fig. 1a). Using simulated libraries with a varied proportion of endogenous DNA, we recover these secondary spikes below 30 bp (Fig. 1b) and find that these spikes derive from bacterial-derived spurious mappings, which is exacerbated at low endogenous DNA contents (Fig. 1c; Supplementary Data S3). Importantly, a conservative MQ threshold of ≥30 greatly reduces the mismatch rate at ultrashort read lengths but only marginally improves the fraction of mappings that are endogenous (Fig. 1b and c). This suggests that caution should be taken, and that appropriate MQ cut-offs should be used, if incorporating ultrashort reads into downstream analyses.

### 3.3 Assessing mapping strategies

**Figure 2.**
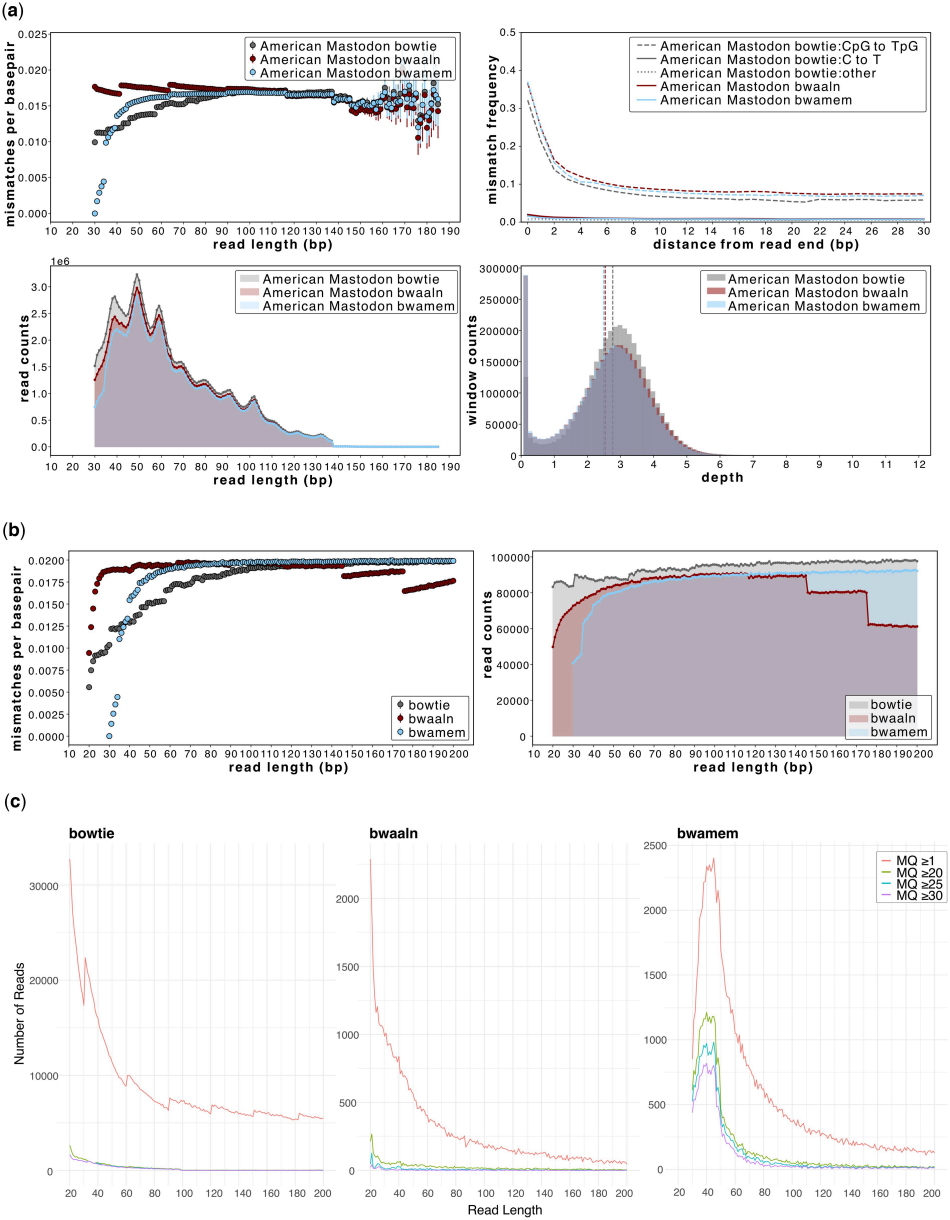
The impact of three mapping algorithms (*BWA-aln*, *Bowtie2*, *BWA-mem*) on ancient genomic datasets. (a) AMBER plots for the American mastodon empirical dataset with MQ ≥1; (b) mismatch and fragment length distribution plots for a simulated dataset of 100% endogenous elephant with 2% sequence divergence at MQ ≥1. (c) The counts of mismapped reads for each read length for the three aligners at MQ ≥1, ≥20, ≥25, or ≥30. For all comparisons, see Supplementary Data S4. *Bowtie2* exhibits the greatest reference bias for read lengths typical of ancient DNA (30–80 bp), whereas *BWA-aln* shows reference bias for read lengths >120 bp. *BWA-mem* does not exhibit this latter bias, but maximizes reference bias for alignments ≤40 bp. There were 100 000 available reads per length bin in all simulated datasets

A wide range of mapping software and aDNA-specific mapping parameters to optimize sequence yield have been explored (Schubert *et al.* 2012, Martiniano *et al.* 2020, Poullet and Orlando 2020, Oliva *et al.* 2021). The optimal mapping parameters are often sample-specific, and dependent on the reference genome used, the type of samples being analysed, and the questions under investigation. A commonly used statistic for assessing optimal mapping strategies is the mapped read count, which disregards the effects of spurious mappings and reference bias. AMBER allows the evaluation of mapping strategies and how they affect the underlying data, helping the user to implement the optimal strategy for their specific case. We used AMBER to compare USER-treated sequencing reads of an American mastodon that was mapped to the Asian elephant genome using *BWA-aln*, *BWA-mem* and *Bowtie2*, and filtered with MQ ≥1. In this particular example, a ∼10 bp periodicity is also observed in the fragment length distribution, consistent with DNA fragmentation in the presence of histone-DNA complexes (Pedersen *et al.* 2014). To further explore the three alignment tools, we additionally used simulated elephant reads with 2% sequence divergence, which is comparable to the observed divergence between American mastodon and Asian elephant (Fig. 2a, top left). In both the empirical and simulated data, *Bowtie2* resulted in the greatest proportion of mapped data (Fig. 2a and b). At short read lengths (30–40 bp), *BWA-mem* displays a precipitous increase in reference bias, *Bowtie2* recovers only two-thirds of the expected mismatches per bp, whereas *BWA-aln* shows performance comparable to other read lengths (Fig. 2a and b, top left). However, between ∼40 and 80 bp, which includes the majority of empirical data, we observe that reads mapped with *Bowtie2* have fewer mismatches to the reference than those mapped with *BWA-aln* and *BWA-mem* (Fig. 2a and b, top left). This implies that the divergence between American mastodon and Asian elephant would be significantly underestimated when analysing a *Bowtie2* mapped genome. This reference bias is mirrored in the aDNA damage plot, whereby *Bowtie2*-mapped data appears to exhibit less damage than the *BWA*-mapped data (Fig. 2a, top right). In contrast, a step-down pattern, or pull to the reference, is observed at longer read lengths (>120 bp) with *BWA-aln* although this appears to not affect those mapped with *BWA-mem* (Fig. 2b, right). The read mismapping rate of simulated data is lower for both *BWA-aln* and *BWA-mem* as compared to *Bowtie2* (Fig. 2c). However, the *Bowtie2* mismapping rate is greatly reduced at MQ ≥20 (Fig. 2c, Supplementary Data S4).

### 3.4 The impact of map quality filtering thresholds

**Figure 3.**
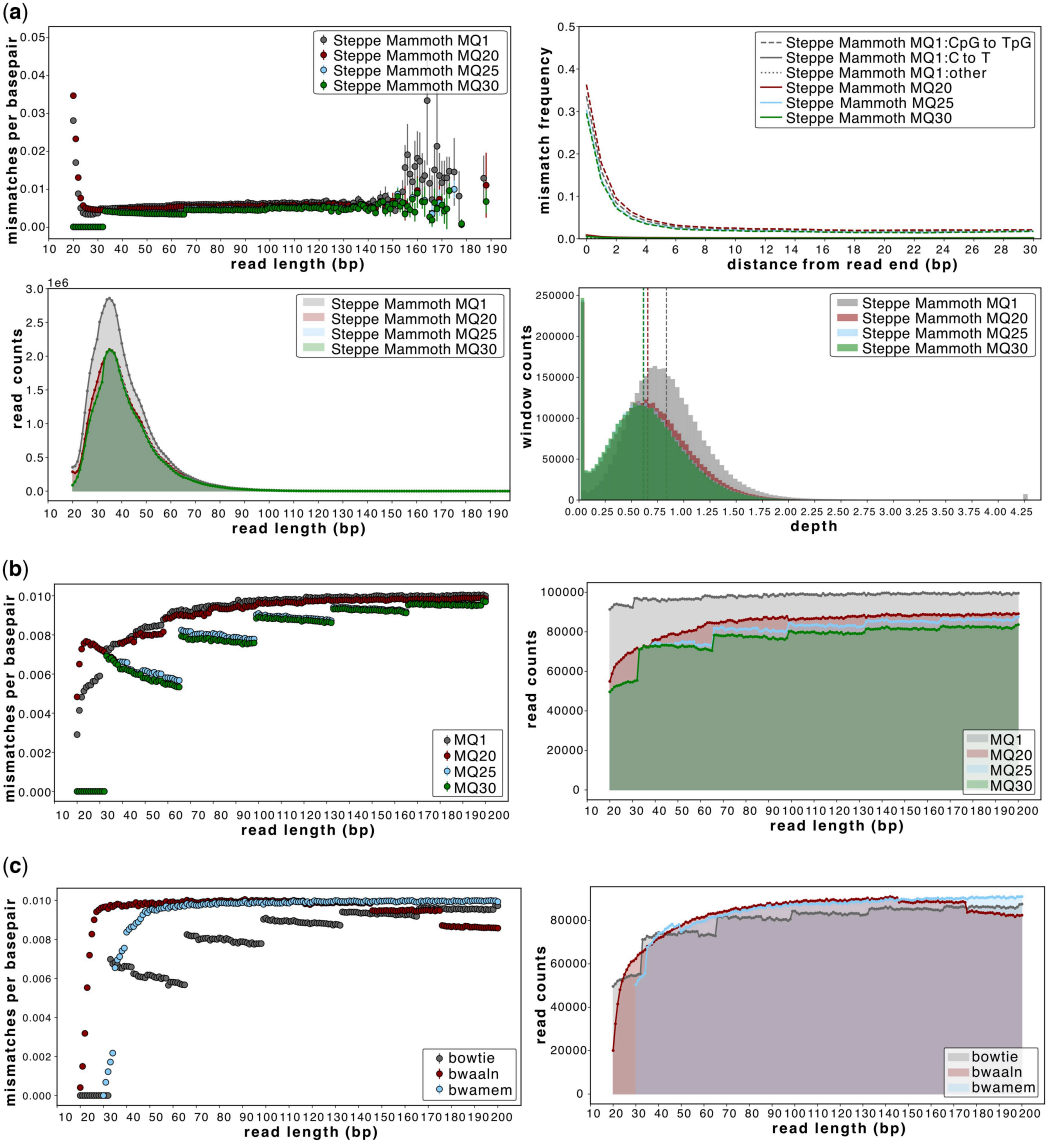
The impact of filtering using different MQ thresholds (≥1, ≥20, ≥25, or ≥30) on ancient genomic datasets. (a) AMBER plots for the steppe mammoth empirical dataset mapped with *Bowtie2*; (b) mismatch and fragment length distribution plots for a simulated dataset of 100% endogenous Asian elephant reads with 1% sequence divergence mapped with *Bowtie2* at varying MQ thresholds; (c) *Bowtie2*, *BWA-aln* and *BWA-mem*, and using MQ ≥25. Higher MQ score thresholds differentially impact the various aligners, with the greatest impact on *Bowtie2*-mapped data. There were 100 000 available reads per length bin in all simulated datasets. The vertical dashed lines on panel (a) indicate the average depth of coverage achieved for each MQ threshold considered

After mapping, filtering of reads based on a minimum MQ threshold is commonly employed. Such filters may differentially affect reads of varying lengths. Using AMBER, we compared both the *Bowtie2*-mapped steppe mammoth and simulated elephant at 1% divergence datasets filtered for increasing MQ filters (≥1, ≥20, ≥25, ≥30). In both the empirical and simulated examples, we show that a strict MQ filter of ≥25 results in a strong reference bias (Fig. 3a and b, top left) that is most pronounced in the shortest reads. Using MQ ≥1, reads below 65 bp are ∼50% more divergent from the reference compared to using MQ ≥30. Crucially, below a read length of ∼32 bp, this bias at MQ ≥30 only retains reads without any mismatches despite the known divergence between the sample and reference. As with the mapping software comparison (Fig. 2a and b), the usage of different MQ filters also impacts deamination profile estimates (Fig. 3a, top right). Although we focus on *Bowtie2* here, we note that the calculation of MQ scores differs between aligners and that this can affect downstream comparisons of datasets using different alignment tools if the same MQ filters are applied (Fig. 3c). A threshold of up to MQ 25 only marginally impacts *BWA-aln* mapping results, whereas MQ ≥30 generates a reference bias across all read lengths (Supplementary Text S4).

### 3.5 Assessing the effect of reference-sample edit distance

**Figure 4.**
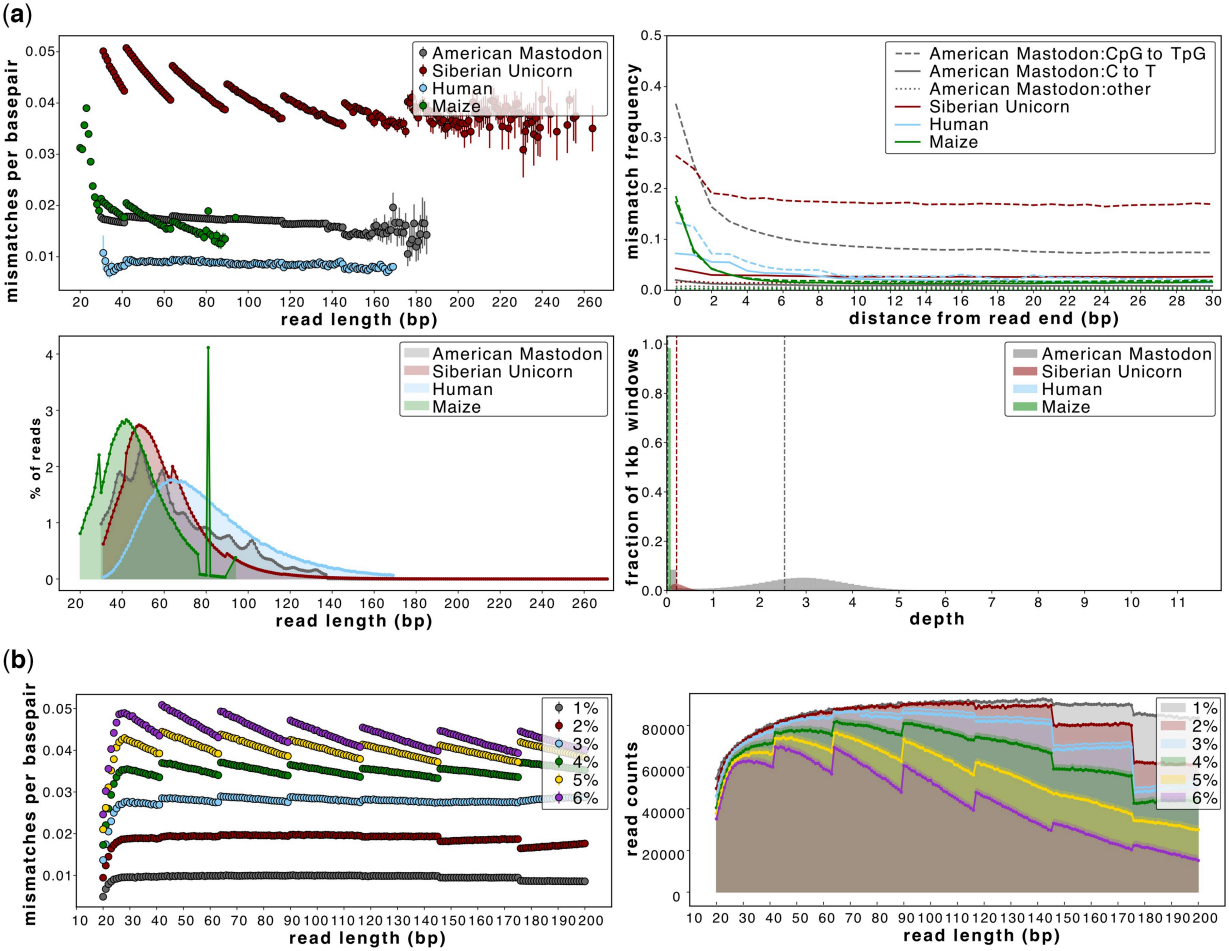
The impact of ancient genomic datasets with differing sample-reference edit distances (sequence divergence). (a) AMBER plots for a non-USER-treated Siberian unicorn, a USER-treated American mastodon, and non-USER-treated maize and human mapped with *BWA-aln* and MQ ≥1; (b) mismatch and fragment length distribution plots for simulated datasets of 100% endogenous elephant reads with 1%–6% sequence divergence mapped with *BWA-aln* and MQ ≥1. Higher sequence divergences, especially those ≥3%, are greatly impacted by reference bias. Comparisons of up to 15% sequence divergence and a USER/non-USER comparison can be found in Supplementary Texts S5 and S6. There were 100 000 available reads per length bin in all simulated datasets. The vertical dashed lines on panel (a) indicate the average depth of coverage achieved in each species

Since reference genomes are unavailable for extinct species, interspecific reference genomes are used for mapping aDNA data. This can result in a substantial genetic distance between reads and the reference, especially if the sample has no close living relative, is of deep-time age (e.g. >100 000 years old), and/or has high levels of aDNA damage. Increased genetic distance leads to increased reference bias, which can differentially impact reads of varying lengths. AMBER provides a means to observe sample-reference divergence and to roughly quantify and evaluate the extent of reference bias in ancient genomes (Fig. 4). We show that reference bias is most pronounced when the genetic distance between a sample and reference is extreme, as exemplified by the non-USER treated Siberan unicorn, and increasingly in the simulated datasets with a divergence of >3% (Fig. 4a and b, top left). Reference bias is less pronounced when the sample-reference divergence is lower, as is the case for the USER-treated American mastodon, and simulated datasets with 1%–3% divergence (Fig. 4). However, we show that even at the lowest sample-reference divergence, as is the case for human and simulated datasets with 1% divergence, reference bias can be pronounced in long reads (>145 bp), which is likely due to the limitations of *BWA-aln* with aDNA parameters (Fig. 4a and b).

## 4 Discussion

AMBER fills a gap in the toolkit for aDNA data authentication by providing a diversity of read mapping quality checks for up to six genomes simultaneously. AMBER allows the evaluation of data integrity prior to downstream analyses while offering users the chance to increase their data yield by allowing ultrashort reads to be included in the dataset. We anticipate that AMBER will aid discovery of bioinformatic biases introduced during data processing, such as the effects of different read trimming and merging tools, alternative mapping algorithms, parameters, and MQ filters not investigated here, and the resultant read length biases. AMBER could also reveal novel biases of which the aDNA community is not yet aware, especially now that the field is moving towards ever older, more degraded, and low endogenous samples (van der Valk *et al.* 2021, Kjær *et al.* 2022). Given its speed, AMBER can also be used to confirm that AMBER-informed improvements to sample data processing have been successful, by determining the best-fit reference genome to mitigate reference bias. We anticipate that this feature will gain increasing popularity as the number of reference genomes available through pan-genome initiatives increases. Nonetheless, aDNA samples are highly complex, and even with the statistics provided by AMBER, nonendogenous data can mimic expected aDNA patterns and look authentic. We therefore recommend using AMBER only as a first data authentication check. Other tools offer additional features, such as the assessment of post-mortem depurination footprints in mapDamage2 (Jónsson *et al.* 2013), and so we advocate the use of multiple tools in cases where additional detailed quality control checks are desired. We further emphasize that study-specific quality checks should also be employed during further downstream analyses such as calling or imputing variants.

Reference bias and spurious mapping remain a significant concern in aDNA research, especially as datasets push the boundaries of alignment software tools not intended for use on such diverged, deaminated, and fragmented reads. These phenomena can occur when mapping to either a conspecific or closely related genome in which the alignment tool favours alleles found within the reference over alternative alleles (Günther and Nettelblad 2019, Orlando *et al.* 2021). The findings of this study demonstrate that reference bias and spurious mapping are dependent on factors such as sequence length and divergence, alignment tool, and MQ filtering.

Within the mapping parameters considered in this study, it appears that *Bowtie2* has a stronger reference bias than *BWA-aln* or *BWA-mem* at aDNA-relevant read lengths (≤80 bp), especially with MQ filter thresholds of ≥20. To mitigate this issue, one approach could be to use MQ ≥1; however, this retains many mismapped reads across all aligners with the rate of mismapping being an order of magnitude higher with *Bowtie2* than the two *BWA* options (Fig. 2; Supplementary Data S4). The higher rate of mismapping seen with *Bowtie2* compared to *BWA* has previously been noted (Hatem *et al.* 2013), including at higher MQ filter thresholds of up to 30 (Poullet and Orlando 2020). However, our simulations reveal that at longer read lengths (>100 bp), the *Bowtie2* mismapping rate is negligible when MQ ≥20. Further, a previous benchmarking study found that *BWA-aln* outperforms *BWA-mem* for aDNA data, likely due to its limitations at shorter read lengths (Oliva *et al.* 2021). Therefore, we recommend that a combination of both *BWA-aln* for shorter reads and *BWA-mem* or *Bowtie2* for longer reads can mitigate the step-down pattern, or reference bias, seen in *BWA-aln* mappings of longer reads while maintaining the higher accuracy for shorter reads observed with *BWA-aln*. We emphasize that the threshold between ‘shorter’ and ‘longer’ reads is dependent on sample-reference sequence divergence but can be inferred from an AMBER mismatch plot. For example, in the simulated datasets with 100% endogenous DNA, it is recommended to switch to *BWA-mem* at >100 bp read lengths. Future efforts to mitigate reference and spurious mapping biases could focus on mapping reads using a third-allele reference, which allows both alleles at heterozygous sites to be considered equally when mapping, therefore greatly reducing the impact of reference bias, albeit at the cost of decreased mapping rates (Günther and Nettelblad 2019, Vernot *et al.* 2021).

While AMBER can be used to observe reference bias and spurious mappings to understand data biases and inform sample-specific read length cutoffs, in addition to biological relevant inferences such as genomic sex determination (Supplementary Text S2), a deeper understanding of the impact of reference bias on downstream population genomics analyses is integral. For example, a previous study using principal components analysis found that samples were separated on the PC2 axis by read length rather than population (Meisner *et al.* 2021), which could have led to misinformed interpretations. These previous observations and our findings suggest that read length biases warrant further research for their impact on downstream analyses, including DNA methylation mapping, and especially those reliant on random allele sampling.

## Supplementary Material

btae436_Supplementary_Data

## Data Availability

AMBER was tested on publicly available datasets, with data accession details given in Supplementary Table S2. Reference genome accessions are given in Sections 2.2.2 and 2.2.3.

## References

[btae436-B1] Briggs AW , Stenzel U, Johnson PLF et al Patterns of damage in genomic DNA sequences from a neandertal. Proc Natl Acad Sci USA 2007;104:14616–21. 17715061 10.1073/pnas.0704665104 PMC1976210

[btae436-B2] Briggs AW , Stenzel U, Meyer M et al Removal of deaminated cytosines and detection of in vivo methylation in ancient DNA. Nucleic Acids Res 2010;38:e87. 20028723 10.1093/nar/gkp1163 PMC2847228

[btae436-B3] Chen S , Zhou Y, Chen Y et al fastp: an ultra-fast all-in-one FASTQ preprocessor. Bioinformatics 2018;34:i884–90. 30423086 10.1093/bioinformatics/bty560 PMC6129281

[btae436-B4] Fernandez-Guerra A , Borrel G, Delmont TO et al A 2-million-year-old microbial and viral communities from the Kap København Formation in North Greenland. bioRxiv, 10.1101/2023.06.10.544454, 2023, preprint: not peer reviewed.

[btae436-B5] Feuerborn TR , Palkopoulou E, van der Valk T et al Competitive mapping allows for the identification and exclusion of human DNA contamination in ancient faunal genomic datasets. BMC Genomics 2020;21:844. 33256612 10.1186/s12864-020-07229-y PMC7708127

[btae436-B6] de Filippo C , Meyer M, Prüfer K et al Quantifying and reducing spurious alignments for the analysis of ultra-short ancient DNA sequences. BMC Biol 2018;16:121. 30359256 10.1186/s12915-018-0581-9 PMC6202837

[btae436-B7] Green RE , Krause J, Briggs AW et al A draft sequence of the Neandertal genome. Science 2010;328:710–22. 20448178 10.1126/science.1188021 PMC5100745

[btae436-B8] Günther T , Nettelblad C. The presence and impact of reference bias on population genomic studies of prehistoric human populations. PLoS Genet 2019;15:e1008302. 31348818 10.1371/journal.pgen.1008302 PMC6685638

[btae436-B9] Hatem A , Bozdağ D, Toland AE et al Benchmarking short sequence mapping tools. BMC Bioinformatics 2013;14:184. 23758764 10.1186/1471-2105-14-184 PMC3694458

[btae436-B10] Heger A , Belgrad TG, Goodson M et al pysam: Python interface for the SAM/BAM sequence alignment and mapping format. 2014. https://github.com/pysam-developers/pysam.

[btae436-B11] Hunter JD. Matplotlib: a 2D graphics environment. Comput Sci Eng 2007;9:90–5.

[btae436-B12] Jónsson H , Ginolhac A, Schubert M et al mapDamage2.0: fast approximate Bayesian estimates of ancient DNA damage parameters. Bioinformatics 2013;29:1682–4. 23613487 10.1093/bioinformatics/btt193 PMC3694634

[btae436-B13] Kjær KH , Winther Pedersen M, De Sanctis B et al; PhyloNorway Consortium. A 2-million-year-old ecosystem in Greenland uncovered by environmental DNA. Nature 2022;612:283–91. 36477129 10.1038/s41586-022-05453-y PMC9729109

[btae436-B14] Langmead B , Salzberg SL. Fast gapped-read alignment with Bowtie 2. Nat Methods 2012;9:357–9. 22388286 10.1038/nmeth.1923 PMC3322381

[btae436-B15] Li H. Aligning sequence reads, clone sequences and assembly contigs with BWA-MEM. arXiv, arXiv:1303.3997 [q-bio.GN], 2013, preprint: not peer reviewed.

[btae436-B16] Li H , Handsaker B, Wysoker A et al; 1000 Genome Project Data Processing Subgroup. The sequence alignment/map format and SAMtools. Bioinformatics 2009;25:2078–9. 19505943 10.1093/bioinformatics/btp352 PMC2723002

[btae436-B17] Li H , Durbin R. Fast and accurate short read alignment with Burrows–Wheeler transform. Bioinformatics 2009;25:1754–60. 19451168 10.1093/bioinformatics/btp324 PMC2705234

[btae436-B18] Martiniano R , Garrison E, Jones ER et al Removing reference bias and improving indel calling in ancient DNA data analysis by mapping to a sequence variation graph. Genome Biol 2020;21:250. 32943086 10.1186/s13059-020-02160-7 PMC7499850

[btae436-B19] Meisner J , Albrechtsen A, Hanghøj K et al Detecting selection in low-coverage high-throughput sequencing data using principal component analysis. BMC Bioinformatics 2021;22:470. 34587903 10.1186/s12859-021-04375-2 PMC8480091

[btae436-B20] Oliva A , Tobler R, Cooper A et al Systematic benchmark of ancient DNA read mapping. Brief Bioinform 2021;22:bbab076. 10.1093/bib/bbab076 33834210

[btae436-B21] Orlando L , Allaby R, Skoglund P et al Ancient DNA analysis. Nat Rev Methods Primers 2021;1:1–26.

[btae436-B22] Pedersen JS , Valen E, Velazquez AMV et al Genome-wide nucleosome map and cytosine methylation levels of an ancient human genome. Genome Res 2014;24:454–66. 24299735 10.1101/gr.163592.113 PMC3941110

[btae436-B23] Pochon Z , Bergfeldt N, Kırdök E et al aMeta: an accurate and memory-efficient ancient metagenomic profiling workflow. Genome Biol 2023;24:242. 37872569 10.1186/s13059-023-03083-9 PMC10591440

[btae436-B24] Poullet M , Orlando L. Assessing DNA sequence alignment methods for characterizing ancient genomes and methylomes. Front Ecol Evol 2020;8:105.

[btae436-B25] Renaud G , Hanghøj K, Willerslev E et al Gargammel: a sequence simulator for ancient DNA. Bioinformatics 2017;33:577–9. 27794556 10.1093/bioinformatics/btw670 PMC5408798

[btae436-B26] Sawyer S , Krause J, Guschanski K et al Temporal patterns of nucleotide misincorporations and DNA fragmentation in ancient DNA. PLoS One 2012;7:e34131. 22479540 10.1371/journal.pone.0034131 PMC3316601

[btae436-B27] Schubert M , Ginolhac A, Lindgreen S et al Improving ancient DNA read mapping against modern reference genomes. BMC Genomics 2012;13:178. 22574660 10.1186/1471-2164-13-178 PMC3468387

[btae436-B28] Skoglund P , Northoff BH, Shunkov MV et al Separating endogenous ancient DNA from modern day contamination in a Siberian Neandertal. Proc Natl Acad Sci USA 2014;111:2229–34. 24469802 10.1073/pnas.1318934111 PMC3926038

[btae436-B29] van der Valk T , Pečnerová P, Díez-Del-Molino D et al Million-year-old DNA sheds light on the genomic history of mammoths. Nature 2021;591:265–9. 33597750 10.1038/s41586-021-03224-9 PMC7116897

[btae436-B30] Vernot B , Zavala EI, Gómez-Olivencia A et al Unearthing Neanderthal population history using nuclear and mitochondrial DNA from cave sediments. Science 2021;372:abf1667. 10.1126/science.abf1667 33858989

[btae436-B95864] Xu W, , Lin YU, , Zhao K et al An efficient pipeline for ancient DNA mapping and recovery of endogenous ancient DNA FROM whole‐genome sequencing data. Ecology and Evolution 2021;11:390–401. 10.1002/ece3.7056. 33437437 PMC7790629

