# Supplementary material for: Unravelling reference bias in ancient DNA datasets
Source: Bioinformatics. 2024 Jul 3;40(7):btae436. doi: 10.1093/bioinformatics/btae436 (PMC11254355; doi:10.1093/bioinformatics/btae436)
Supplement: btae436_Supplementary_Data [file btae436_supplementary_data.zip › AMBER Supplementary Document.pdf]

Unravelling reference bias in ancient DNA datasets

Stephanie Dolenz[1,2,#], Tom van der Valk[1,3,4,#], Chenyu Jin[1,3,5], Jonas Oppenheimer[6], Bilal Sharif[1,5], Ludovic Orlando[7], Beth Shapiro[8,9], Love Dalén[1,3,5], Peter D. Heintzman[1,2]

1. Centre for Palaeogenetics, Stockholm, Sweden
2. Department of Geological Sciences, Stockholm University, Stockholm, Sweden
3. Department of Bioinformatics and Genetics, Swedish Museum of Natural History, Stockholm, Sweden
4. Science for Life Laboratory, Stockholm, Sweden
5. Department of Zoology, Stockholm University, Stockholm, Sweden
6. Department of Biomolecular Engineering, University of California Santa Cruz, Santa Cruz, CA, USA
7. Centre for Anthropobiology and Genomics of Toulouse (CAGT, CNRS UMR5288), University Paul Sabatier, Faculté de Santé, 31000 Toulouse, France
8. Department of Ecology and Evolutionary Biology, University of California Santa Cruz, Santa Cruz, CA, USA
9. Howard Hughes Medical Institute, University of California Santa Cruz, Santa Cruz, CA, USA

[#]*joint first authors: stephanie.dolenz@geo.su.se, tom.vandervalk@nrm.se*

Supplementary Information

Text S1. Assessing ancient metagenomic data **2**

Figure S1. Two microbial genomes derived from an ancient metagenomic sample. 3

Text S2. Sex determination and mitochondrial-nuclear genome comparison **4**

Figure S2. Genomic compartments of the American mastodon mapped to the Asian elephant genome using Bowtie2 and MQ ≥20. 4

Text S3. Tool run-time comparison **5**

Table S1. Run-time comparison of commonly used ancient DNA evaluation tools. 5

Text S4. Mapping quality filter thresholds across all three aligners **6**

Figure S3. The impact of mapping quality filter thresholds across all three aligners. 6

Text S5. Reference bias under extreme divergence scenarios **7**

Figure S4. Reference bias at extreme sequence divergence scenarios. 7

Text S6. USER and non-USER treatment comparison **8**

Figure S5. The impact of USER treatment on a horse palaeogenome. 8

Text S7. Comparison of different Bowtie2 mapping parameters **9**

Figure S6. A comparison of different mapping parameters for Bowtie2 on empirical and simulated ancient genomic datasets. 10

Table S2. Empirical ancient DNA datasets used to evaluate AMBER and investigate reference bias. 11

Data S1. AMBER plots for all simulated Asian elephant data combinations **12**

Data S2. AMBER plots for all simulated Black rhinoceros data combinations **12**

Data S3. Read origin plots for all simulated data combinations **12**

Data S4. Mapping statistics plots for all simulated data combinations **12**

Supplementary References **13**

Text S1. Assessing ancient metagenomic data

We classified all sequence reads from the ancient metagenomic sample (Table 1) against the microbial Genome Taxonomy Database (GTDB; June-2023 release) using Kraken2 v2.1.2 on default settings with the --report minimizer-data parameter (Wood *et al.*, 2019). We searched the Kraken2 output for a bacterial genome that showed a high number of assigned minimizers but few unique minimizers (a likely false hit) and a bacterial genome with both a high number of total and unique minimizers (a likely true hit). We aligned all kraken classified sequence reads to these two respective bacterial reference genomes (GTDB IDs SOKP01_sp019894735 and Lutibacter_sp016649745, respectively) using *Bowtie2* v2.3.5.1 (Langmead and Salzberg, 2012) with the --sensitive setting.

In ancient metagenomic data analysis, spurious mappings and modern DNA contamination can give a false indication of species presence. The summary statistics provided by AMBER allow users to filter for ancient taxa inferred to be truly present from taxa that are likely false positives. We used AMBER to analyze two micro-organism genomes that were observed in a 2 million year old sediment sample from Greenland (Kjær *et al.*, 2022; Fernandez-Guerra *et al.*, 2023); belonging to the *Candidatus Lokiarchaeota* archaeon and *Flavobacteriaceae* bacterium groups respectively. Both taxa had a high number of mapped reads (75,256 and 1,497,322 respectively) but AMBER shows that the *Flavobacteriaceae* bacterium genome does not display any signs of aDNA damage (Figure S1, top right). Whereas the *Flavobacteriaceae* bacterium genome coverage is on average 25× (Figure S1, bottom right), a large fraction (~14%) of the genome remains completely uncovered. The low mismatch rates to the reference show that *Flavobacteriaceae* bacterium mapped reads are closely related to the modern reference sample, which is highly unlikely for a microbe that is 2 million years old. Overall, these observations suggest that the *Flavobacteriaceae* bacterium genome observed in the sample is likely a modern contaminant. In contrast, a high rate of aDNA damage and normally-distributed depth across the genome is observed for the *Candidatus Lokiarchaeota* archaeon genome. Together with the higher mismatch rate to the reference (the observed stochasticity is due to the relatively low number of mapped sequence reads), this suggests that the *Candidatus Lokiarchaeota* archaeon genome represents an ancient microbe that is truly present in the sample.

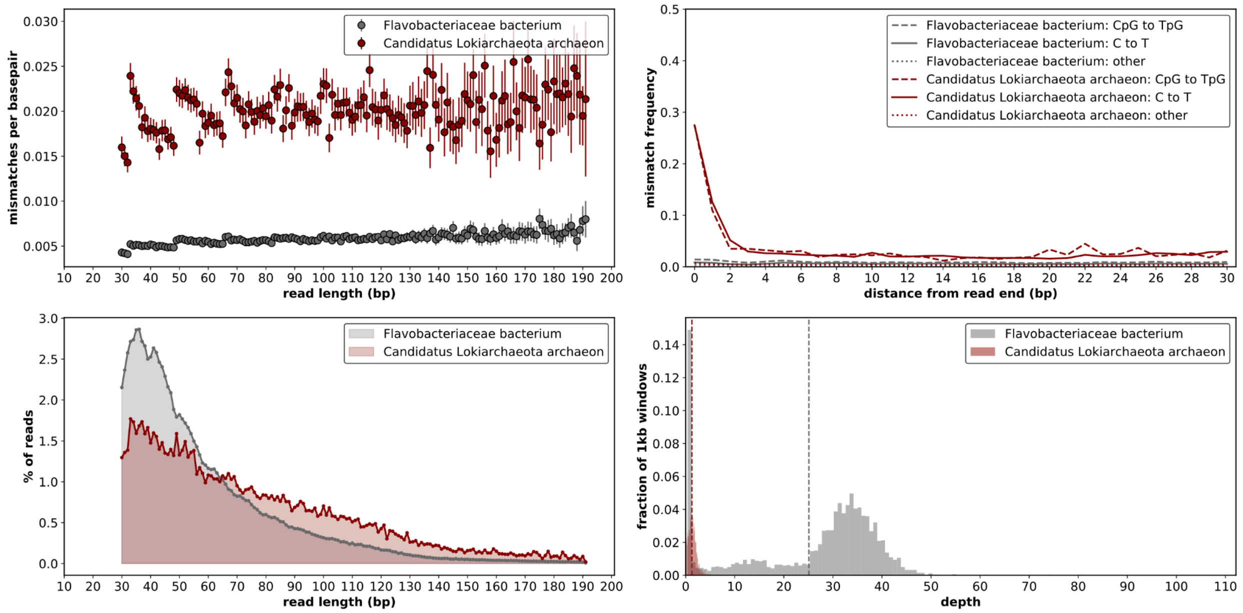

Figure S1. Two microbial genomes derived from an ancient metagenomic sample.

The sample reads are aligned to the respective reference genomes of *Candidatus Lokiarchaeota* archaeon and *Flavobacteriaceae* bacterium using *Bowtie2* and MQ ≥20.

Text S2. Sex determination and mitochondrial-nuclear genome comparison

The rate of molecular evolutionary processes varies across genomic regions. For instance, comparative analyses among mammals have revealed a higher degree of sequence conservation in X chromosome sequences compared to autosomes (Ellegren, 2011). In contrast, the mitogenome exhibits an accelerated evolutionary rate relative to the nuclear genome in animals, whereas it is relatively conserved in plants (Yang *et al.*, 1998; Lynch *et al.*, 2006). Furthermore, DNA degradation can potentially differentially impact various regions of the genome. For example, in contrast to the autosomes, CpG sites within mitogenomes remain consistently unmethylated thus not displaying a characteristic aDNA damage pattern when samples are USER treated (Figure S2, top right). These variations in evolutionary and preservation mechanisms may thus introduce mapping biases within the same sample across distinct genomic compartments (Figure S2, top left). AMBER facilitates the identification and visualization of these biases within a sample by allowing users to separately analyze specific genomic regions (Figure S2). Importantly, AMBER can be used to estimate the genomic sex of individuals. For example, in the American mastodon individual processed through AMBER, it can be deduced that this individual is genomically male, based on the lower coverage of the X chromosome, which is almost half that of the autosomes (Figure S2, lower right). AMBER therefore enables the separation of differential mapping biases across distinct genomic compartments within a sample in addition to the ability to rapidly analyze the genomic sex of an individual.

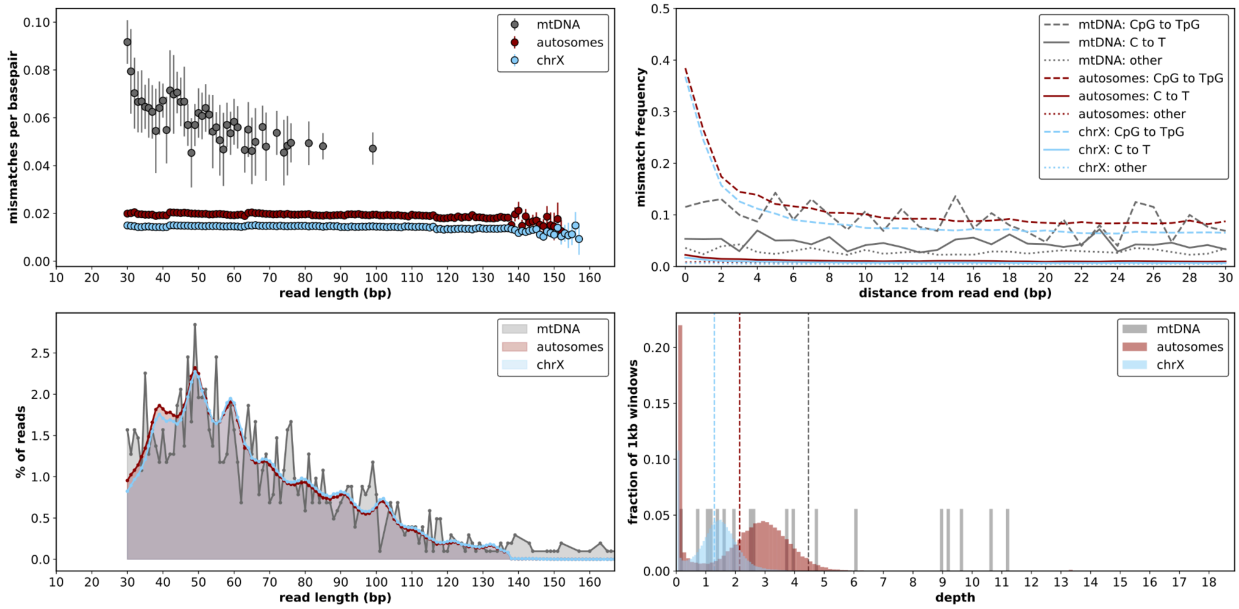

Figure S2. Genomic compartments of the American mastodon mapped to the Asian elephant genome using *Bowtie2* and MQ ≥20.

The genome BAM-file was divided into three separate BAM files based on genomic compartments (autosomes, X-chromosome, mitogenome), which were then analyzed in AMBER.

Text S3. Tool run-time comparison

Although no ancient DNA software provides all four BAM-file quality checks that are implemented in AMBER simultaneously, each of the assessments is available as separate tools or scripts. We compared the running time of AMBER against some of the most commonly used tools that assess mismatches per bp (van der Valk *et al.*, 2021), fragment length distribution (Jónsson *et al.*, 2013), and post-mortem DNA damage either genome-wide (Jónsson *et al.*, 2013) or specifically at CpG sites (Skoglund, Northoff, *et al.*, 2014) (Table S1). With the exception of the *readLenCutoff* tool provided in (van der Valk *et al.*, 2021), the running time of AMBER is up to an order of magnitude faster than other currently available softwares.

Table S1. Run-time comparison of commonly used ancient DNA evaluation tools.

**Tool**	**Reference**	**Running time per million reads**	**DNA damage (all C to T)**	**DNA damage (CpG to TpG)**	**Fragment length distribution**	**Reference mismatch rate**
mapDamage v2.2.2	(Jónsson *et al.*, 2013)	499 sec.	*		*	
PMDtools v0.60	(Skoglund, Northoff, *et al.*, 2014)	7215 sec.	*	*		
readLengthCutoff * (only SAM files)	(van der Valk *et al.*, 2021)	23 sec.				*
***AMBER* v1.0**	**This study**	**72 sec.**	*****	*****	*****	*****

Available functions are given with asterisks. Wall clock time was evaluated on a single core on the Intel Xeon V4 CPU. Asterisks indicate tool functionality.

Text S4. Mapping quality filter thresholds across all three aligners

We compared the impact of mapping quality (MQ) threshold choice across the three tested aligners: *Bowtie2*, *BWA-aln*, and *BWA-mem* (Figure S3). We find that *Bowtie2* is the most impacted of the three aligners by MQ threshold choice, with any MQ exhibiting reference bias in shorter reads (<100 bp), although there is a minimal impact when increasing the minimum MQ threshold from 25 to 30. In contrast, *BWA-aln* is most impacted when the minimum MQ threshold is increased from 25 to 30, with all read lengths exhibiting an increase in reference bias. At MQ ≤25, *BWA-aln* robustly maps short reads (30-100 bp) with minimal reference bias, although this bias increases in a stepwise fashion at longer read lengths (>120 bp). *BWA-mem* mappings are almost unaffected by MQ threshold choice. We emphasize that these results should also be interpreted in the context of mis-mapping rate, which is greatest for *BWA-mem* (e.g. Main text, Figure 2c; Data S4).

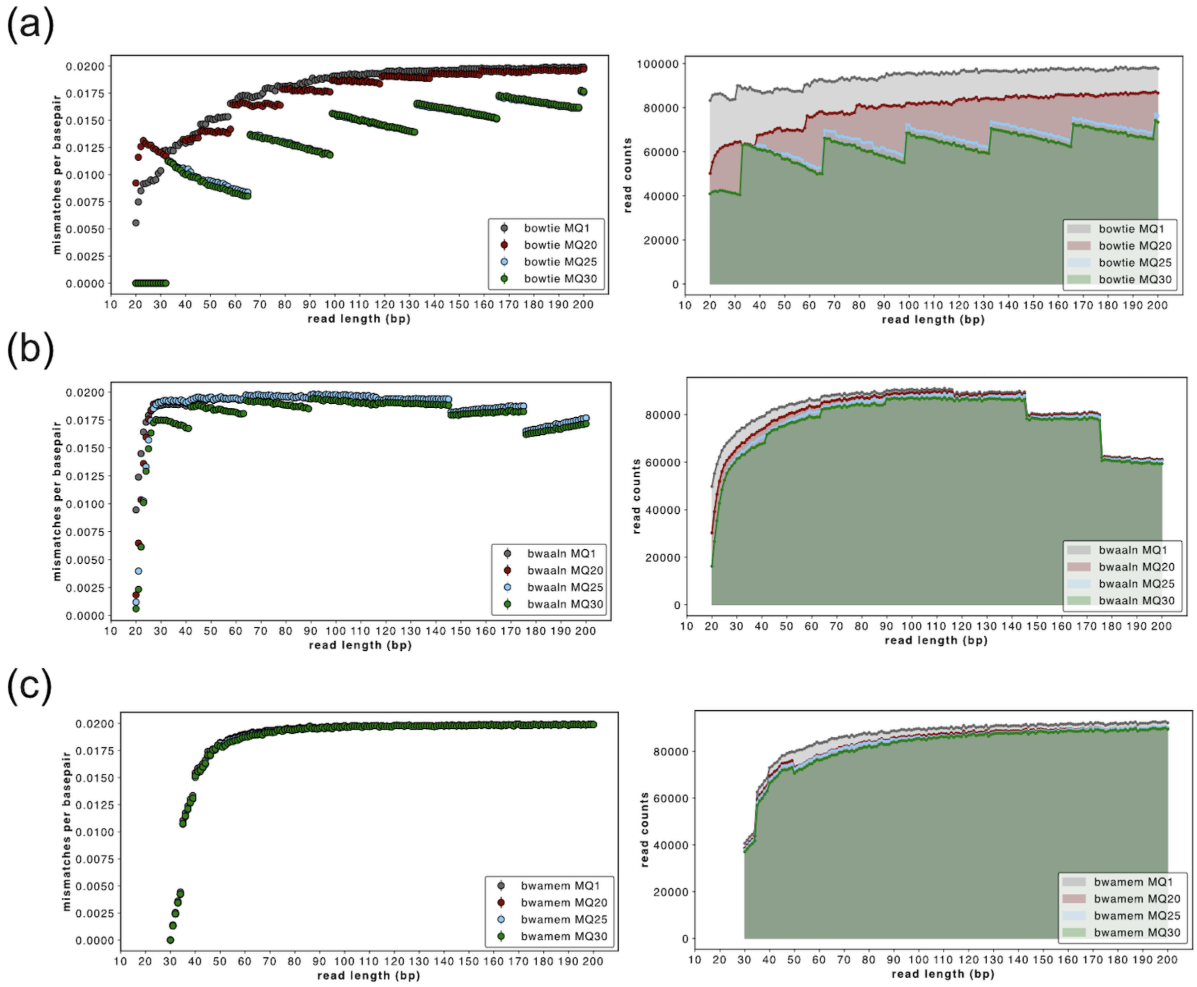

Figure S3. The impact of mapping quality filter thresholds across all three aligners.

A comparison of four MQ thresholds (≥1, ≥20, ≥25, or ≥30) on a simulated elephant dataset with 2% sequence divergence that have been mapped with (a) *Bowtie2*, (b) *BWA-aln*, and (c) *BWA-mem*. There were 100,000 available reads per length bin in all simulated datasets. Comparisons across all sequence divergences can be found in Data S1 and S2.

Text S5. Reference bias under extreme divergence scenarios

We simulated sequence divergences up to 15% to explore how reference bias is impacted under extreme divergence scenarios and test the limits of the three tested aligners. We find that for reads of typical aDNA length (40-80 bp), *Bowtie2* can map reads up to a maximum of ~5-6% mean divergence, *BWA-aln* up to ~6-8%, and *BWA-mem* up to ~4-10% (Figure S4). However, we note that mis-mappings also become increasingly common, and mapping rates decline, as divergence increases (Figure S4; Data S4). These scenarios highlight the challenges of mapping to reference genomes that are extremely diverged from the sample.

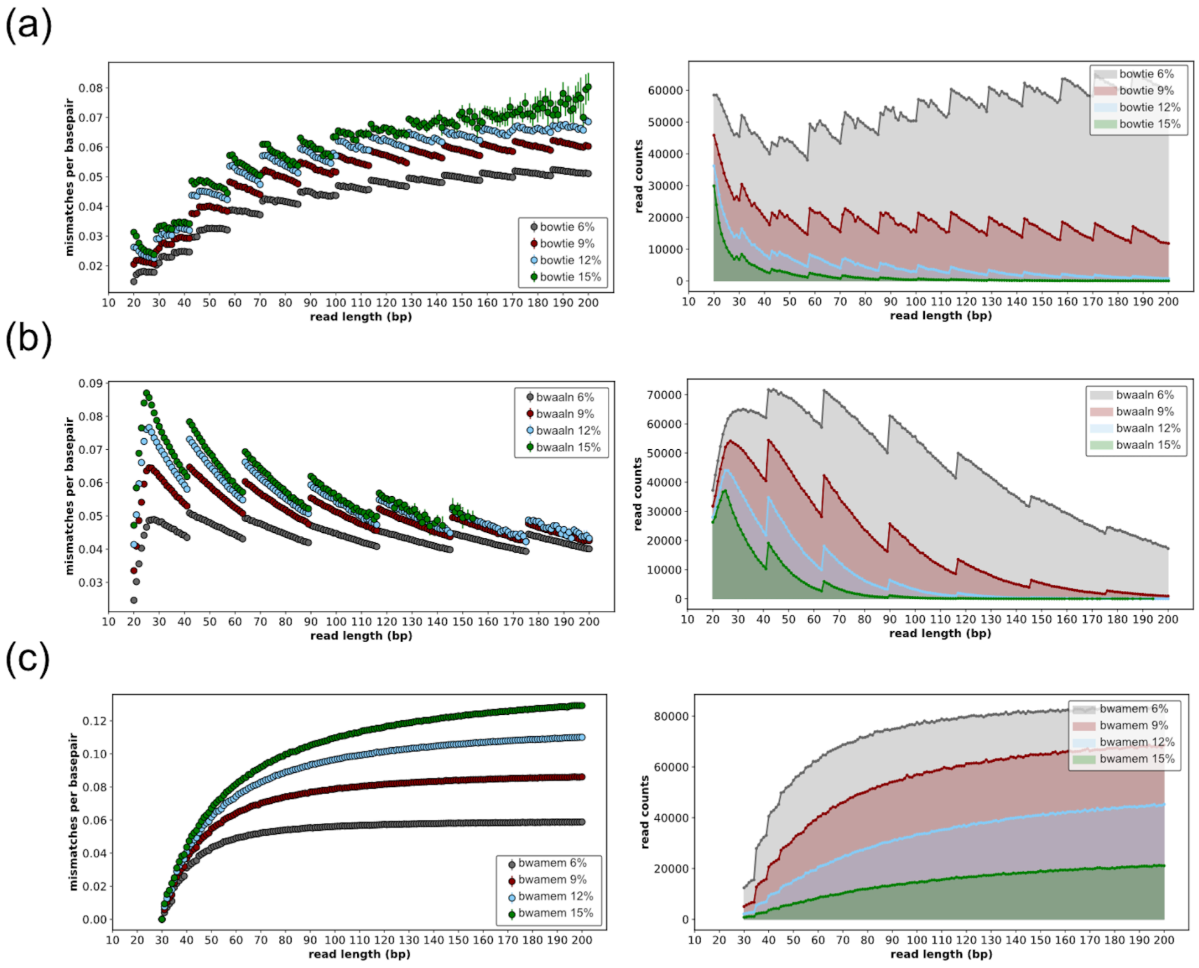

Figure S4. Reference bias at extreme sequence divergence scenarios.

Simulated elephant reads with sequence divergences of 6%, 9%, 12%, and 15%, mapped at MQ ≥1 with (a) *Bowtie2*, (b) *BWA-aln*, and (c) *BWA-mem*. There were 100,000 available reads per length bin in all simulated datasets.

Text S6. USER and non-USER treatment comparison

USER treatment is often used to remove cytosine deamination damage at non-CpG and unmethylated-CpG sites (Briggs *et al.*, 2010; Wagner *et al.*, 2020). We used AMBER to observe the difference in ancient horse libraries constructed from the same sample that was constructed either with or without USER treatment (Table 1; Figure S5). As expected from the removal of most aDNA damage, USER treatment greatly reduces the sample-reference divergence across all read lengths, in this case by around two-thirds (Figure S5, top left), and clearly removes damage at non-CpG sites, including those throughout the length of the aDNA molecules (Figure S5, top right).

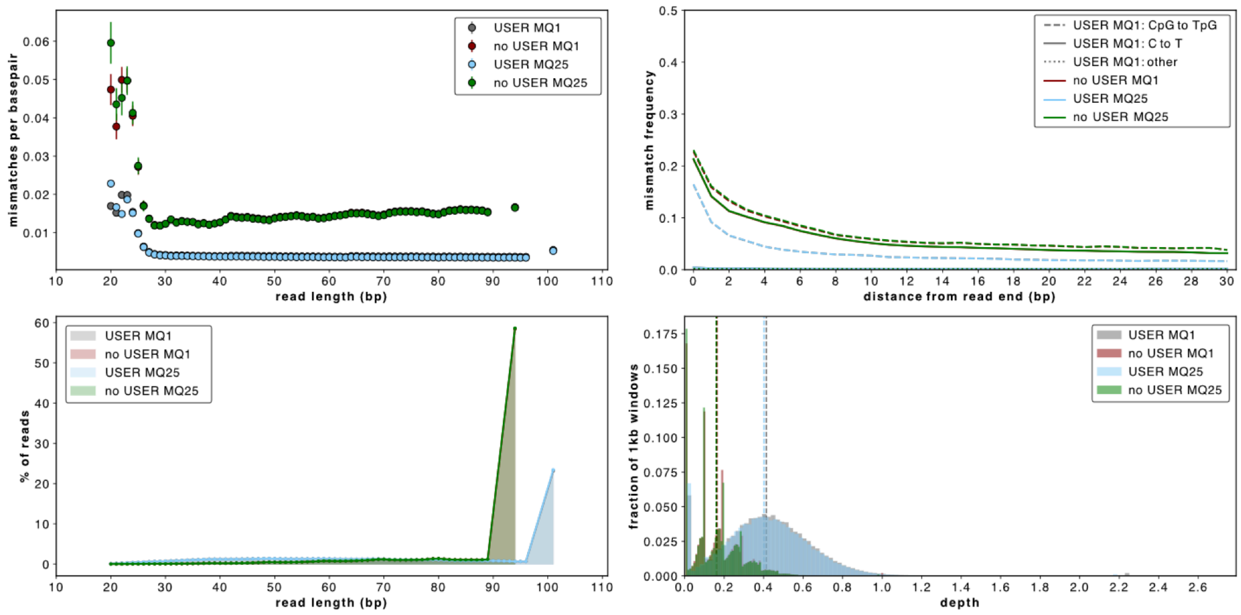

Figure S5. The impact of USER treatment on a horse palaeogenome.

A USER- and non-USER treated library of an ancient horse mapped using *BWA-aln* and with an MQ of either ≥1 or ≥25. The non-USER-treated library carries >3 times as many mismatches per base pair as a USER-treated library.

Text S7. Comparison of different *Bowtie2* mapping parameters

There are two *Bowtie2* alignment modes: *end-to-end*, which uses the entire read for mapping, and *local*, where the read ends can be “clipped” to increase their mappability. In addition, mapping sensitivity can be selected with either the *--very fast*, *--fast*, *--sensitive*, or *--very sensitive* options (Langmead and Salzberg, 2012). Previous studies of alignment tools on palaeogenomic data have compared *Bowtie2 end-to-end* and *local* modes under the different sensitivity options (Oliva *et al.*, 2021; Poullet and Orlando, 2020), with *Bowtie2 end-to-end --sensitive* recommended for USER-treated data and *local* for potential use with non-USER-treated data (Poullet and Orlando, 2020). Using empirical USER-treated steppe mammoth and simulated elephant data at 1% divergence, we find that there is minimal difference between *end-to-end --sensitive* and *--very-sensitive* (Figure S6a,b), with *--very-sensitive* exhibiting a slightly reduced reference bias at shorter read lengths. Furthermore, we show that *local --sensitive* has a lower mapping rate and increased reference bias compared to either of the two tested *end-to-end* options (Figure S6b), despite the mismapping rate reaching 0 at shorter read lengths (~68 bp) for *local --sensitive* compared to either of the tested *end-to-end* options (~100 bp) at MQ ≥20 (Figure S6c). Therefore, when using *Bowtie2* for aligning palaeogenomic sequencing reads to a reference, either *end-to-end --sensitive* or *--very-sensitive* can be used.

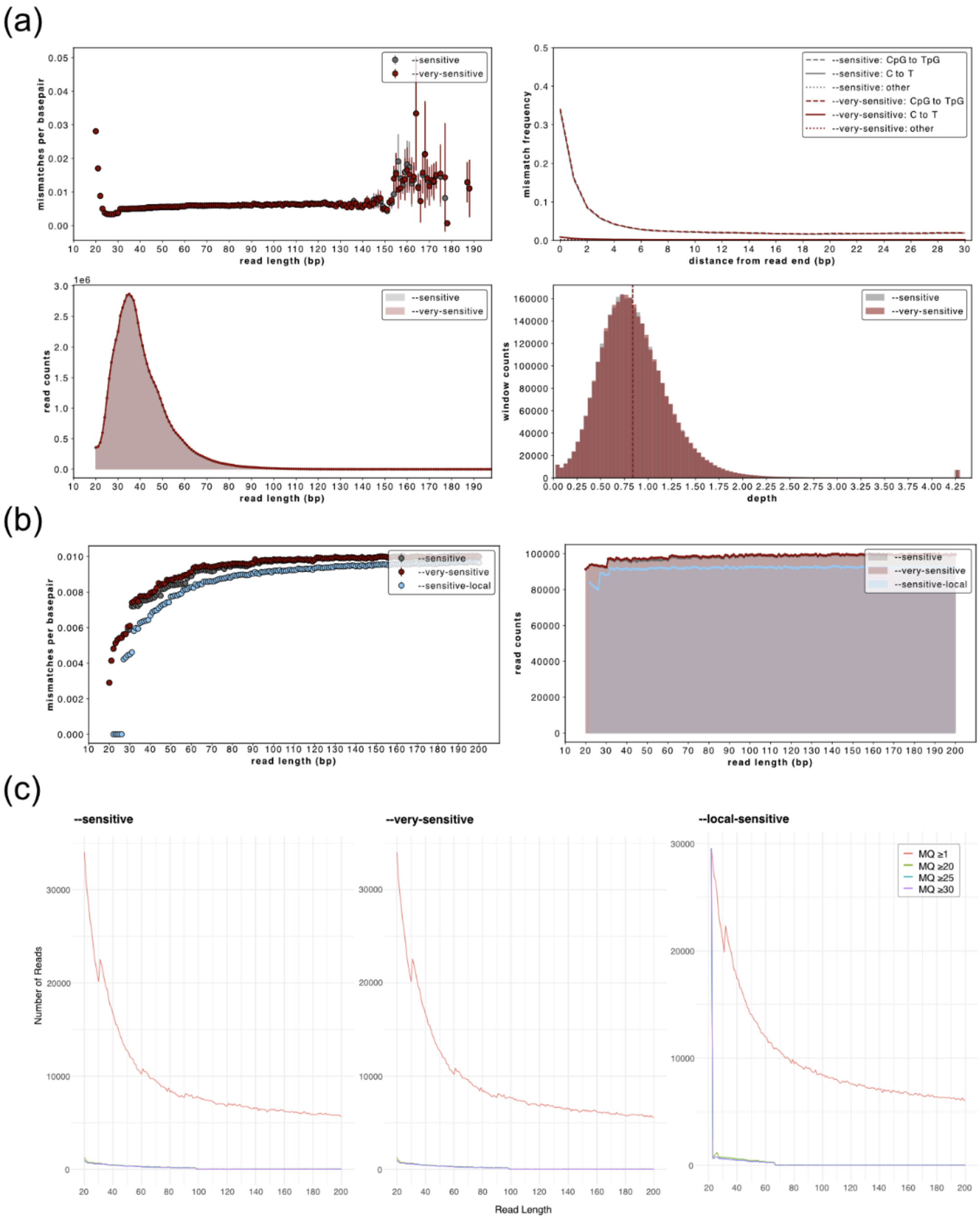

Figure S6. A comparison of different mapping parameters for *Bowtie2* on empirical and simulated ancient genomic datasets.

(a) AMBER plots for the steppe mammoth empirical dataset using *Bowtie2 --sensitive* or *--very-sensitive* with MQ ≥1; (b) Mismatch and fragment length distribution plots for a simulated dataset of 100% endogenous elephant with 1% sequence divergence using all three tested *Bowtie2* mapping parameters at MQ ≥1. (c) The counts of mismapped reads for each read length for the three parameters at MQ ≥1 (red), ≥20 (green), ≥25 (blue), or ≥30 (purple).

Table S2. Empirical ancient DNA datasets used to evaluate AMBER and investigate reference bias.

Sample name	Taxon	Sample age (years)	USER treatment	Reference genome	Input sequence reads	Endogenous content	Genome coverage	Reference	ENA sample accession
PIN 3723-511 (Adycha)	Steppe mammoth (*Mammuthus trogontherii*)	~1.1 Ma	Yes	Interspecific (Asian elephant, 5 Ma)	~2,353 million	~3.8%	~0.3x	(van der Valk *et al.*, 2021)	SAMEA7589371
IK-99-237 (I)	American mastodon (*Mammut americanum*)	~50-150 ka	Yes	Interspecific (Asian elephant, 19 Ma)	~170 million	~42.7%	~4.0x	(Palkopoulou *et al.*, 2018)	SAMEA104469193
IPAE 915/2804	Siberian Unicorn (*Elasmotherium sibiricum*)	>49 ka	No	Interspecific (Black rhinoceros, 36 Ma)	~1,160 million	~25.7%	~9.0x	(Liu *et al.*, 2021)	SAMN17169536
Ajvaide 53 (ACAGTG)	Human (*Homo sapiens*)	~4.8 ka	No	Conspecific (Human)	~35 million	~2.6%	<1x	(Skoglund, Malmström, *et al.*, 2014)	SAMEA2464312
BER06_F_E10.Dab2nd_NOuser_CAGCTA / BER06_F_SCY2.2_user_CGCTAT	Horse (*Equus caballus*)	~2.3 ka	Yes / No	Conspecific (Horse)	~103 million / ~181 million	~45% / ~46%	~1.7x / ~2.3x	(Librado *et al.*, 2017)	SAMEA103910515
Tehuacan162	Maize (*Zea mays*)	~5.3 ka	No	Conspecific (Maize)	~642 million	~70%	~1.7×	(Ramos-Madrigal *et al.*, 2016)	SAMN05977577
119_B3_116_L0_KapK-12-1-27_Ext-4_Lib-4	Ancient sediment (metagenomic)	~2.0 ma	No	Interspecific	~508 million	Not applicable	Not applicable	(Fernandez-Guerra *et al.*, 2023)	SAMEA112130955

Time to the most recent common ancestor for interspecific genome mappings are given as million years ago (Ma). Sample ages are given in either Ma or thousand years ago (ka). Reference genome versions are given in the main text Methods.

Data S1. AMBER plots for all simulated Asian elephant data combinations

(provided as a separate zip file)

Data S2. AMBER plots for all simulated Black rhinoceros data combinations

(provided as a separate zip file)

Data S3. Read origin plots for all simulated data combinations

(provided as a separate zip file)

Data S4. Mapping statistics plots for all simulated data combinations

(provided as a separate zip file)

Supplementary References

Briggs,A.W. *et al.* (2010) Removal of deaminated cytosines and detection of in vivo methylation in ancient DNA. *Nucleic Acids Res.*, **38**, e87.

Ellegren,H. (2011) Sex-chromosome evolution: recent progress and the influence of male and female heterogamety. *Nat. Rev. Genet.*, **12**, 157–166.

Fernandez-Guerra,A. *et al.* (2023) A 2-million-year-old microbial and viral communities from the Kap København Formation in North Greenland. *bioRxiv*, 2023.06.10.544454.

Jónsson,H. *et al.* (2013) mapDamage2.0: fast approximate Bayesian estimates of ancient DNA damage parameters. *Bioinformatics*, **29**, 1682–1684.

Kjær,K.H. *et al.* (2022) A 2-million-year-old ecosystem in Greenland uncovered by environmental DNA. *Nature*, **612**, 283–291.

Langmead,B. and Salzberg,S.L. (2012) Fast gapped-read alignment with Bowtie 2. *Nat. Methods*, **9**, 357–359.

Librado,P. *et al.* (2017) Ancient genomic changes associated with domestication of the horse. *Science*, **356**, 442–445.

Liu,S. *et al.* (2021) Ancient and modern genomes unravel the evolutionary history of the rhinoceros family. *Cell*, **184**, 4874–4885.e16.

Lynch,M. *et al.* (2006) Mutation pressure and the evolution of organelle genomic architecture. *Science*, **311**, 1727–1730.

Oliva,A. *et al.* (2021) Systematic benchmark of ancient DNA read mapping. *Brief. Bioinform.*, **22**.

Palkopoulou,E. *et al.* (2018) A comprehensive genomic history of extinct and living elephants. *Proc. Natl. Acad. Sci. U. S. A.*, **115**, E2566–E2574.

Poullet,M. and Orlando,L. (2020) Assessing DNA Sequence Alignment Methods for Characterizing Ancient Genomes and Methylomes. *Frontiers in Ecology and Evolution*, **8**.

Ramos-Madrigal,J. *et al.* (2016) Genome Sequence of a 5,310-Year-Old Maize Cob Provides Insights into the Early Stages of Maize Domestication. *Curr. Biol.*, **26**, 3195–3201.

Skoglund,P., Malmström,H., *et al.* (2014) Genomic diversity and admixture differs for Stone-Age Scandinavian foragers and farmers. *Science*, **344**, 747–750.

Skoglund,P., Northoff,B.H., *et al.* (2014) Separating endogenous ancient DNA from modern day contamination in a Siberian Neandertal. *Proc. Natl. Acad. Sci. U. S. A.*, **111**, 2229–2234.

van der Valk,T. *et al.* (2021) Million-year-old DNA sheds light on the genomic history of mammoths. *Nature*, **591**, 265–269.

Wagner,S. *et al.* (2020) Uncovering Signatures of DNA Methylation in Ancient Plant Remains From Patterns of Post-mortem DNA Damage. *Frontiers in Ecology and Evolution*, **8**.

Wood,D.E. *et al.* (2019) Improved metagenomic analysis with Kraken 2. *Genome Biol.*, **20**, 257.

Yang,Z. *et al.* (1998) Models of amino acid substitution and applications to mitochondrial protein evolution. *Mol. Biol. Evol.*, **15**, 1600–1611.
